# A LASSO-Based Nomogram for Predicting Focal Complications in Brucellosis: A Multicenter Retrospective Cohort Study

**DOI:** 10.3390/jcm15062180

**Published:** 2026-03-12

**Authors:** Enes Dalmanoğlu, Sevda Ozdemir Al, Ünsal Bağın

**Affiliations:** 1 Department of Infectious Diseases and Clinical Microbiology, Balıkesir University Faculty of Medicine, 10145 Balıkesir, Türkiye; 2 Department of Infectious Diseases and Clinical Microbiology, Tokat Turhal State Hospital, 60300 Tokat, Türkiye; ozdsevda@gmail.com; 3 Department of Infectious Diseases and Clinical Microbiology, Artvin State Hospital, 08000 Artvin, Türkiye; baginunsal@gmail.com

**Keywords:** brucellosis, nomogram, LASSO, prognostic nutritional index, focal complication, prediction model, decision curve analysis, TRIPOD

## Abstract

**Background**: Up to one-third of brucellosis patients develop focal organ involvement, contributing to increased morbidity and therapeutic failure, yet no clinically validated instrument exists to stratify risk at presentation. **Methods**: In this three-center retrospective cohort from Türkiye (2015–2025), 355 adults with confirmed brucellosis were enrolled. Thirty-two candidate variables spanning demographics, comorbidities, symptoms, routine laboratory values, and composite inflammation indices underwent LASSO-penalized regression with 10-fold cross-validation for predictor selection, after which a nomogram was constructed and internally validated via 1000-iteration bootstrap resampling. **Results**: Ninety-two patients (25.9%) developed focal complications. Five predictors were retained by LASSO—prognostic nutritional index (PNI), erythrocyte sedimentation rate (ESR), C-reactive protein (CRP), chronic disease stage, and hypertension—and combined with age and sex (retained a priori) into a seven-predictor nomogram. PNI was the strongest contributor (OR = 0.901, 95% CI: 0.857–0.948). Apparent C-statistic reached 0.782 (optimism-corrected 0.762), with a calibration slope of 0.894 and Brier score of 0.154. Decision curve analysis indicated net clinical benefit over the 5–55% threshold probability range. **Conclusions**: This PNI-anchored LASSO nomogram offers a practical bedside risk stratification instrument for brucellosis-related focal involvement. Prospective external validation across geographically diverse endemic regions is warranted before clinical adoption.

## 1. Introduction

Brucellosis ranks among the most widespread zoonoses worldwide, with over half a million new cases reported annually; the disease is particularly concentrated in the Mediterranean region, the Middle East, and Central Asia [1]. Organ-specific (focal) manifestations—spondylitis, sacroiliitis, peripheral arthritis, orchitis, neurobrucellosis, endocarditis, and abscess formation—develop in roughly 10–30% of affected individuals and constitute the leading drivers of prolonged illness, therapeutic failure, and healthcare expenditure [2,3,4,5].

Notwithstanding improvements in antimicrobial regimens, 5–15% of patients experience treatment failure and 5–40% relapse, with rates being highest in those harboring focal disease [6,7,8]. Earlier investigations have linked several factors—chronicity, diagnostic delay, male sex, advanced age, elevated ESR, thrombocytosis, arthralgia, bacteremia, and suboptimal initial therapy—to an increased likelihood of focal involvement [4,5,9,10,11,12]; nonetheless, these predictors have been examined in isolation rather than combined into a formal risk estimation tool. A bedside instrument capable of identifying high-risk individuals early could guide selective imaging, intensified surveillance, and tailored therapeutic approaches, thereby curtailing avoidable morbidity [13].

Over the past decade, composite biomarker indices calculated from standard hematological and biochemical panels have gained traction as prognostic instruments across diverse clinical settings. Indices such as the neutrophil-to-lymphocyte ratio (NLR), platelet-to-lymphocyte ratio (PLR), systemic immune–inflammation index (SII), systemic inflammation response index (SIRI), Prognostic Nutritional Index (PNI), and CRP-to-albumin ratio (CAR) condense information on immune activation, nutritional reserve, and inflammatory load into single numerical scores [14,15]. Among these, PNI—derived from serum albumin and peripheral lymphocyte count—has independently predicted all-cause mortality in pulmonary tuberculosis [16] and COVID-19 [17]. Investigating such indices in brucellosis is biologically well motivated—*Brucella* species are facultative intracellular organisms that evade T-lymphocyte surveillance and disrupt monocyte–macrophage function, generating an inflammatory milieu that composite indices may capture more comprehensively than any single parameter [3,5,18,19].

Demonstrating biomarker associations alone, however, does not translate into clinical utility. Although earlier work has linked individual inflammatory markers to disease severity in brucellosis, no study to date has synthesized these markers within a multivariable prediction framework for focal complications. A structured literature search across PubMed/MEDLINE, Scopus, and Web of Science returned no validated prediction model or scoring system for this endpoint. The unmet need, therefore, is a bedside-applicable instrument—preferably a nomogram—that fuses several informative predictors into an individualized probability estimate. The current investigation advances beyond single-marker association studies to construct and validate such a composite prognostic tool, following the TRIPOD reporting framework [20].

To address this gap, we designed a multicenter study with three objectives: (1) to employ LASSO regression for data-driven selection of the most informative predictors from 32 candidate variables encompassing demographics, comorbidities, clinical features, and seven inflammation-based indices; (2) to develop a nomogram for individualized prediction of focal complications; and (3) to internally validate the model using bootstrap resampling and evaluate its clinical utility through decision curve analysis (DCA), adhering to the TRIPOD (Transparent Reporting of a multivariable prediction model for Individual Prognosis Or Diagnosis) guidelines.

## 2. Materials and Methods

### 2.1. Study Design, Setting, and Ethics

This retrospective cohort investigation enrolled patients from three Turkish institutions: Balıkesir University Hospital (coordinating site), Tokat Turhal State Hospital, and Artvin State Hospital, covering the period from January 2015 to December 2025. The enrollment pathway is summarized in Supplementary Figure S1. The protocol was approved by the Balıkesir University Faculty of Medicine Health Research Ethics Committee (Approval No: 2026/2-1, Date: 20 January 2026). Informed consent was waived given the retrospective design. The investigation adhered to the principles of the Declaration of Helsinki and was reported in line with the STROBE [21] and TRIPOD [20] checklists (see Supplementary Materials). All three centers followed institutional protocols consistent with Turkish national brucellosis guidelines, in which imaging evaluation (spinal MRI for suspected spondylitis, scrotal ultrasound for orchitis, echocardiography for endocarditis) applies to all patients who present with pertinent clinical symptoms irrespective of laboratory parameters.

### 2.2. Study Population

Consecutive adult patients (≥18 years) with confirmed brucellosis were included. Diagnosis required at least one of the following standard criteria [22]: (1) SAT titer of at least 1:160, (2) a positive Coombs anti-*Brucella* antibody test, or (3) recovery of *Brucella* species in blood culture. Exclusion criteria included incomplete records, concurrent malignancy, immunosuppressive therapy, chronic liver disease (Child–Pugh B/C), pregnancy, and hematological malignancies. A formal a priori sample size calculation was not performed; instead, all consecutive eligible patients during the study period were included. Post hoc sample size adequacy was assessed using the Riley et al. (2020) framework [23]: for a binary outcome with 25.9% event fraction, 7 final predictors, target shrinkage factor of 0.90, and target Nagelkerke R^2^ of 0.15, a minimum of roughly 247 participants and at least 62 outcome events were needed. The final dataset of 355 patients with 92 events exceeded both thresholds. With 32 candidate predictors, the screening-stage events-per-variable ratio was 2.9—below the conventional threshold of 10 [24]; however, LASSO penalization substantially reduces the effective model complexity at the optimal λ. The final model retained 7 parameters, yielding 13.1 events per final predictor. The bootstrap-derived calibration slope of 0.894 provides empirical reassurance that overfitting remained within acceptable limits.

### 2.3. Candidate Predictors

Thirty-two candidate predictors were prespecified based on clinical relevance and prior literature: age, sex, residential setting, disease stage (acute [<8 weeks of symptom duration at presentation] vs. chronic [≥8 weeks], classified in accordance with Franco et al. [2]), comorbidities (diabetes, hypertension, cardiovascular disease), presenting symptoms (fever, night sweats, arthralgia, myalgia, fatigue, weight loss), and laboratory parameters (CRP, ESR, albumin, WBC, neutrophil, lymphocyte, monocyte, hemoglobin, platelet, MCV, ALT, AST). Seven inflammation-based biomarker indices were calculated: NLR = neutrophil/lymphocyte; PLR = platelet/lymphocyte; MLR = monocyte/lymphocyte; SII = (neutrophil × platelet)/lymphocyte; SIRI = (neutrophil × monocyte)/lymphocyte; PNI = albumin (g/L) + 5 × lymphocyte (×10^9^/L); CAR = CRP/albumin. Pairwise Pearson correlation coefficients among all 32 candidate predictors were computed prior to model development to characterize the multicollinearity structure (Supplementary Figure S2). High intercorrelations were observed among composite indices and their constituent laboratory parameters (e.g., neutrophil–WBC r = 0.93, SII–NLR r = 0.89, AST–ALT r = 0.74), which informed the post-LASSO variable refinement criteria described in Section 2.5.

### 2.4. Outcome Definition

The primary outcome was focal complication, defined as any organ-specific involvement confirmed by imaging, microbiological, or clinical criteria. Specific diagnostic criteria were as follows: spondylitis and sacroiliitis (MRI-confirmed); peripheral arthritis (clinical findings of joint inflammation with or without imaging confirmation); orchitis/epididymo-orchitis (scrotal ultrasound and/or clinical findings); neurobrucellosis (cerebrospinal fluid analysis with compatible neurological findings [25]); endocarditis (modified Duke criteria); and abscess formation (imaging-confirmed by CT or ultrasound). Focal complications were ascertained both at the time of initial diagnosis and throughout the treatment and follow-up period. Patients presenting with imaging-confirmed focal disease at initial evaluation were classified as having focal complications from baseline, while complications developing during treatment or follow-up were similarly captured. We acknowledge that diagnostic stringency varied across complication types, with peripheral arthritis and orchitis potentially diagnosed on clinical grounds alone. Secondary outcomes were treatment failure (defined as persistent symptoms, rising or unchanged serological titers, or requirement for treatment modification at ≥3 months) and relapse (recurrence of symptoms with serological confirmation after documented clinical and serological cure).

### 2.5. Statistical Analysis

**Variable selection.** Penalized logistic regression employing L1 (LASSO) regularization was fitted with 10-fold stratified cross-validation. Before model fitting, all 32 candidate predictors underwent z-score standardization so that coefficient magnitudes would be directly comparable. The penalty strength was set at the value (λ min) that minimized mean cross-validated deviance; predictors retaining non-zero coefficients at this optimum were carried forward as LASSO-selected. From the LASSO-selected variables, a parsimonious final predictor set was derived using a two-stage approach: LASSO for initial data-driven screening, followed by clinician-guided refinement based on the following prespecified criteria: (a) variables with Pearson |r| ≥ 0.70 with a higher-ranked LASSO variable were removed as redundant (see Supplementary Figure S2); (b) subjective symptom-based variables (myalgia, fatigue, night sweats, weight loss) were excluded given their limited reliability in retrospective chart review; and (c) variables with marginal LASSO coefficients (|β| < 0.10) were excluded. This approach is characterized as LASSO-informed, clinician-refined variable selection rather than purely data-driven selection, as investigator judgment was applied after examining the LASSO output. Age and sex were retained as a priori adjustment covariates regardless of LASSO selection, based on their well-established influence on brucellosis epidemiology, inflammatory biomarker distributions, and disease severity. This follows recommendations by Steyerberg (2019) [26] for including clinically important confounders in prediction models even when not selected by automated methods, to reduce confounding bias and improve model transportability across populations with different age–sex distributions.

**Model development.** Multivariate logistic regression was fitted using LASSO-selected variables plus a priori covariates. Results are reported as odds ratios (OR) with 95% confidence intervals (CI). We note that this two-stage approach (LASSO selection followed by unpenalized refit) may produce confidence intervals and *p*-values that are anti-conservative due to post-selection inference bias; therefore, shrinkage-adjusted odds ratios are also reported, derived by applying the bootstrap-estimated calibration slope (uniform shrinkage factor = 0.894) to the linear predictor coefficients. For completeness, the penalized LASSO coefficient estimates at the optimal λ are also reported in Supplementary Table S1. Discriminatory ability was quantified by the C-statistic. Calibration was appraised using the calibration slope, calibration intercept, observed-to-expected (O:E) ratio, Brier score, a graphical calibration plot, and the Hosmer–Lemeshow goodness-of-fit statistic; however, the Hosmer–Lemeshow test is reported for completeness only, as it is known to be sample-size-dependent and of limited value for prediction model calibration assessment [26]. Multicollinearity was assessed by variance inflation factors (VIF). The nomogram was generated using a custom Python implementation based on the matplotlib library (v3.8), with point assignments derived from the logistic regression coefficients scaled to a 0–100 point range.

**Internal validation.** Optimism in apparent performance metrics was quantified through 1000-fold bootstrap resampling [27]. Within each resample the entire modeling pipeline—LASSO selection followed by logistic regression—was refit, and the gap between in-sample and out-of-sample performance was recorded. Averaging this gap across all resamples yielded the optimism estimate, which was subtracted from apparent C-statistic and calibration slope to obtain bias-corrected values.

**Incremental value assessment.** The nomogram’s additive prognostic contribution over conventional clinical variables was quantified by comparing it with a reference model containing age, sex, chronic stage, hypertension, diabetes, and fever. Three complementary reclassification statistics were computed: the change in area under the curve (ΔAUC), category-free net reclassification improvement (NRI), and integrated discrimination improvement (IDI) [28]. We note that category-free NRI may overestimate reclassification improvement and should be interpreted alongside IDI, which provides a more conservative estimate. Model parsimony was compared using the Akaike Information Criterion (AIC).

**Clinical utility.** The clinical usefulness of the nomogram was appraised through decision curve analysis (DCA) [29], which calculates the net benefit at each threshold probability relative to the default strategies of treating every patient or withholding treatment entirely, and additionally against a univariate PNI-only reference model.

**Sensitivity analysis.** Leave-one-center-out analyses were conducted to assess robustness across centers. All tests were two-tailed (α = 0.05). Analyses used Python 3.12 with scikit-learn 1.5 (LogisticRegressionCV with saga solver), statsmodels 0.14, and matplotlib 3.8.

**Missing data.** Complete-case analysis was the primary approach. Among the 32 candidate predictors, demographic and clinical variables had no missing data. Laboratory parameters were missing in <2% of cases (consistent with routine clinical ordering patterns, assumed missing at random). Blood culture status was missing in 26.5% of patients due to non-uniform ordering practices and was excluded from the candidate predictor set rather than imputed, given the non-random nature of blood culture ordering (preferentially obtained in febrile or acutely ill patients). As a sensitivity analysis, multiple imputation by chained equations (MICE; 20 imputation cycles, 50 iterations per cycle) was carried out, applying predictive mean matching to continuous predictors and logistic regression to dichotomous ones, with all 32 candidate predictors plus the outcome included in the imputation model. Complete-case and MICE-imputed model coefficients were compared to assess the impact of missing data on the final estimates (Supplementary Table S2). MICE convergence was assessed visually by inspecting trace plots of imputed values across iterations; stable convergence was achieved by iteration 20 for all imputed variables (trace plots available upon request).

## 3. Results

### 3.1. Study Population and Cohort Characteristics

The final cohort comprised 355 patients (Balıkesir 181, Tokat 118, Artvin 56), with a mean age of 48.3 ± 15.5 years; 261 (73.5%) were male and 295 (83.1%) lived in rural settings. Animal contact was reported in 284 (80.0%) and raw dairy consumption in 234 (65.9%). Chronic disease stage was present in 37 patients (10.4%). The median follow-up was 12 months (IQR: 12–24).

### 3.2. Focal Complications and Baseline Characteristics

Focal complications occurred in 92 patients (25.9%): spondylitis 44 (47.8%), peripheral arthritis 32 (34.8%), orchitis 18/68 male focal patients (26.5% of males; 19.6% of all focal patients), abscess 8 (8.7%), and meningitis 1 (1.1%). Some patients had more than one focal complication. Patients with focal complications had significantly lower PNI (48.7 ± 5.3 vs. 52.6 ± 5.8, *p* < 0.001), higher CRP (30.8 vs. 18.8 mg/L, *p* < 0.001), higher ESR (30 vs. 20 mm/h, *p* < 0.001), and lower albumin (39 vs. 41 g/L, *p* < 0.001) compared with non-focal patients. All seven biomarker indices differed significantly between groups (all *p* < 0.01; Table 1). In a post hoc exploratory analysis, osteoarticular complications (spondylitis + peripheral arthritis; *n* = 73) and non-osteoarticular complications (orchitis, abscess, neurobrucellosis, endocarditis; *n* = 19) were compared separately against non-focal patients: PNI was significantly lower in both subgroups (osteoarticular: 48.8 ± 5.5 vs. 52.6 ± 5.7, *p* < 0.001; non-osteoarticular: 48.5 ± 4.9 vs. 52.6 ± 5.7, *p* = 0.002), supporting PNI as a general predictor of focal disease rather than a subtype-specific marker. Notably, many of the inflammatory biomarkers that differed significantly between groups are intercorrelated, as composite indices (SII, SIRI, CAR, PNI) are arithmetically derived from overlapping laboratory parameters (see Supplementary Figure S2). This multicollinearity provided the rationale for employing LASSO regression for variable selection.

### 3.3. Lasso Variable Selection

**Figure 1:**
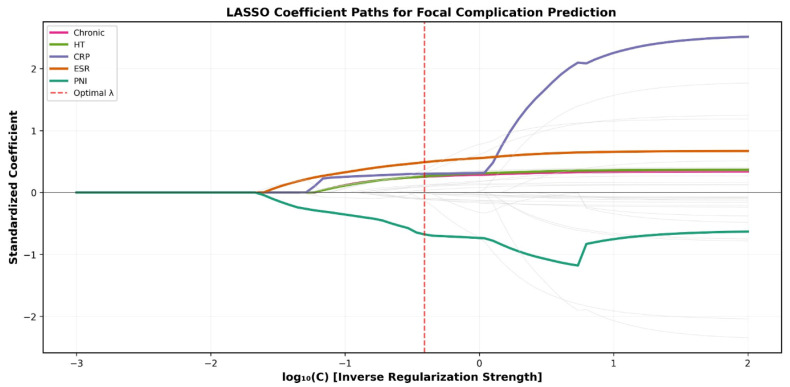
LASSO coefficient paths for 32 candidate predictors of focal complications in brucellosis. Each line represents one variable’s standardized coefficient as a function of regularization strength (log10C). Key variables (PNI, ESR, CRP, chronic stage, hypertension) are highlighted in color; eliminated variables are shown in gray. The vertical dashed red line indicates the penalty parameter chosen via 10-fold cross-validation. C represents the inverse of the regularization parameter λ (C = 1/λ), as implemented in scikit-learn.

LASSO regression applied to 32 standardized candidate predictors identified 22 variables with non-zero coefficients (Figure 1). PNI had the largest absolute standardized coefficient (β = −0.677), followed by ESR (β = 0.489), neutrophil count (β = 0.371), platelet count (β = 0.321), and CRP (β = 0.301). The 10-fold cross-validated AUC of the full LASSO model was 0.760 ± 0.081. Notably, LASSO eliminated NLR, PLR, CAR, SIRI, albumin, and lymphocyte count—variables that were significant in univariate analysis—demonstrating that their prognostic information was captured by PNI and other retained variables, thereby reducing redundancy. The pairwise correlation structure among all 32 candidate predictors is presented in Supplementary Figure S2.

### 3.4. Nomogram Development

**Figure 2:**
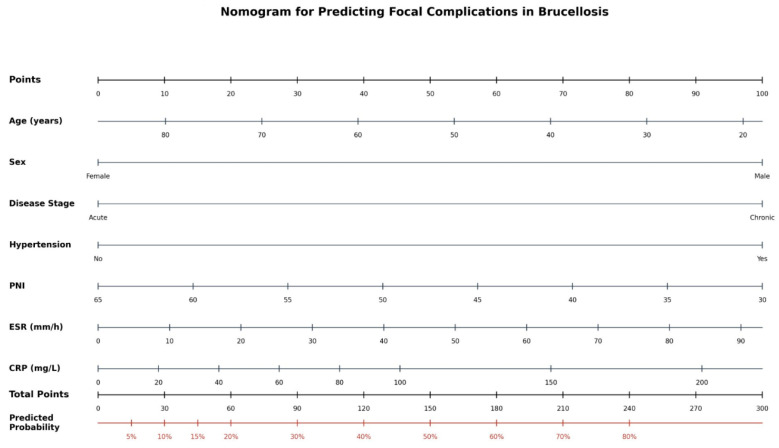
Nomogram for predicting the probability of focal complications in brucellosis. To use: locate the patient’s value on each variable axis, draw a vertical line to the Points axis to determine the points for each variable, sum all points to obtain the Total Points, and project downward to the Predicted Probability axis. The model is based on LASSO-selected variables with bootstrap-validated performance (optimism-corrected C-statistic = 0.762).

From the 22 LASSO-retained variables, a parsimonious model was constructed by applying prespecified criteria: exclusion of variables with high pairwise correlation (|r| ≥ 0.70) with a higher-ranked predictor, removal of subjective symptom-based variables, and exclusion of variables with marginal LASSO coefficients (|β| < 0.10). The complete variable selection results, including retention rationale for each predictor, are detailed in Supplementary Table S1. This process retained five clinical predictors (PNI, ESR, CRP, chronic disease stage, and hypertension) plus two demographic covariates (age and sex) retained a priori, yielding a seven-variable nomogram (Figure 2). The model demonstrated no significant multicollinearity (all VIF < 1.25) (Table 2).

**Figure 3:**
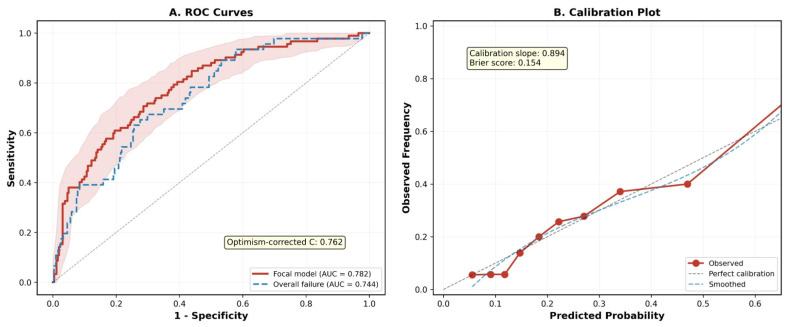
Model performance. (**A**) Receiver operating characteristic (ROC) curves for the focal complication model (red, AUC = 0.782) and overall treatment failure model (blue, AUC = 0.744). The shaded area represents the 95% bootstrap confidence band. (**B**) Calibration plot showing predicted probabilities versus observed frequencies across deciles. The dashed diagonal represents perfect calibration. Calibration slope = 0.894, Brier score = 0.154.

PNI (OR = 0.901 per unit increase, 95% CI: 0.857–0.948, *p* < 0.001): each unit decrease in PNI increased the odds of focal complication by approximately 10%. ESR (OR = 1.030 per mm/h, 95% CI: 1.013–1.047, *p* < 0.001) and CRP (OR = 1.014 per mg/L, 95% CI: 1.005–1.022, *p* = 0.002) contributed independently after mutual adjustment, despite their shared acute-phase biology—their VIF values (CRP: 1.18; ESR: 1.21) confirmed acceptable collinearity, consistent with the known differential kinetics of CRP (rapid response, hepatic synthesis driven by IL-6) versus ESR (slower response reflecting fibrinogen and immunoglobulin levels). Chronic disease stage conferred a 2.5-fold risk (OR = 2.481, *p* = 0.027) consistent with prior multicenter findings [30], and hypertension a 2.1-fold risk (OR = 2.123, *p* = 0.043). The model demonstrated no significant multicollinearity (all VIF < 1.25). A nomogram was constructed for bedside application (Figure 2). The apparent C-statistic was 0.782 (Figure 3A). The corresponding forest plot is presented in Supplementary Figure S3.

Adjusted for age and sex a priori. Apparent C-statistic = 0.782; optimism-corrected C = 0.762 (bootstrap 1000×, percentile CI). Calibration slope = 0.894; calibration intercept = −0.089 (95% CI: −0.40 to 0.30); O:E ratio = 1.00 (95% CI: 0.83–1.21). Hosmer–Lemeshow *p* = 0.857 (reported for completeness; see text for limitations). Brier score = 0.154. VIF: PNI 1.12, ESR 1.21, CRP 1.18, chronic stage 1.08, hypertension 1.05. Shrinkage-adjusted ORs (uniform shrinkage factor = 0.894 applied to log-odds coefficients): PNI 0.911, ESR 1.027, CRP 1.012, chronic stage 2.246, hypertension 1.956, male sex 1.114, age 0.996. Confidence intervals from the unpenalized refit may be anti-conservative due to post-selection inference; shrinkage-adjusted estimates are recommended for external application.

### 3.5. Internal Validation

Bootstrap internal validation (1000 iterations) yielded an optimism of 0.020, resulting in an optimism-corrected C-statistic of 0.762 (95% CI: 0.745–0.780, derived from the 2.5th and 97.5th percentiles of the bootstrap distribution). The calibration slope was 0.894, indicating mild overfitting consistent with expectations for the sample size and number of predictors; this value also serves as the recommended uniform shrinkage factor for future external application. The calibration intercept was −0.089 (95% CI: −0.40 to 0.30), indicating negligible systematic miscalibration. The observed-to-expected (O:E) ratio was 1.00 (95% CI: 0.83–1.21), confirming adequate overall calibration. The Brier score was 0.154. The Hosmer–Lemeshow test was non-significant (*p* = 0.857); however, this test is reported for completeness only, as it is known to be sample-size-dependent and of limited value for prediction model assessment [26]. The calibration plot (Figure 3B) represents apparent (not optimism-corrected) performance, while the calibration slope and optimism-corrected C-statistic derive from the bootstrap procedure. Figure 3A shows the ROC curves for both the focal complication model (AUC = 0.782) and the overall treatment failure model (AUC = 0.744) with 95% CI shading.

### 3.6. Incremental Value over Conventional Model

Compared with the conventional clinical model (age, sex, chronic stage, hypertension, diabetes, fever; AUC = 0.607, AIC = 410.6), the nomogram demonstrated substantial improvement: AUC = 0.782 (ΔAUC = 0.175), AIC = 355.0 (ΔAIC = 55.6), category-free NRI = 0.857 (NRI^events^ = 0.391, NRI^non-events^ = 0.466), and IDI = 0.167. These metrics indicate that the addition of PNI, ESR, and CRP correctly reclassified 85.7% of patients and improved discrimination by 16.7 percentage points on average (Supplementary Table S3). Of note, category-free NRI may overestimate reclassification improvement compared with category-based approaches; the IDI provides a more conservative estimate of incremental value.

### 3.7. Decision Curve Analysis

**Figure 4:**
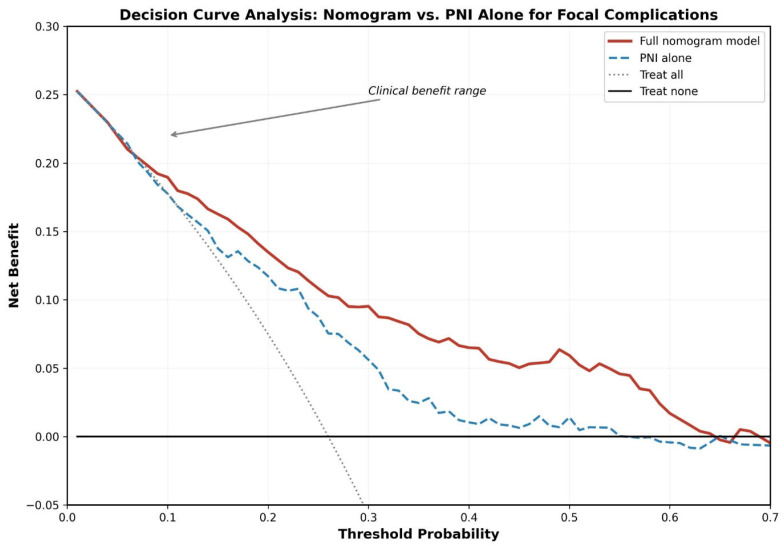
Decision curve analysis comparing the net benefit of the nomogram (red), PNI alone (blue dashed), treat-all strategy (gray dotted), and treat-none strategy (black) across threshold probabilities. The nomogram provides superior net benefit across threshold probabilities of 5–55%, indicating clinical utility for identifying patients who would benefit from enhanced surveillance and treatment intensification.

On DCA (Figure 4), the nomogram yielded a positive net benefit over a broad spectrum of threshold probabilities (5–55%), surpassing the treat-all, treat-none, and PNI-only strategies at every evaluated threshold. The nomogram was particularly advantageous at threshold probabilities of 15–40%, which corresponds to the clinically relevant decision range for initiating enhanced surveillance or treatment intensification.

### 3.8. Risk Stratification

Model-predicted probability tertiles defined three clinically meaningful risk groups: low risk (predicted probability < 12.4%, *n* = 118) with a 6.8% focal complication rate, medium risk (12.4–27.8%, *n* = 119) with a 22.7% rate, and high risk (>27.8%, *n* = 118) with a 48.3% rate (*p*_trend_ < 0.001). The high-risk group had a 7.1-fold higher complication rate compared with the low-risk group (Table 3).

### 3.9. Secondary Outcomes

The following secondary outcome analyses should be interpreted as exploratory and hypothesis-generating, given that the event counts for treatment failure (*n* = 22) and relapse (*n* = 26) preclude reliable multivariable modeling. For treatment failure (*n* = 22, 6.2%), LASSO selected only albumin and PNI, with albumin being the dominant predictor in multivariate analysis (OR = 0.841 per g/L, *p* = 0.003; model AUC = 0.766). For relapse (*n* = 26, 7.3%), chronic disease stage was the dominant predictor (OR = 8.492, *p* < 0.001; model AUC = 0.731). For overall treatment failure (*n* = 46, 13.0%), a three-variable model (PNI, chronic disease stage, ESR) achieved AUC = 0.744 (Figure 3A, blue dashed line). These exploratory findings require confirmation in larger datasets with adequate event counts.

### 3.10. Sensitivity Analysis

Leave-one-center-out analyses confirmed model robustness (Supplementary Table S4). When Balıkesir was excluded (*n* = 174), PNI remained the dominant predictor (OR = 0.895, *p* = 0.002) with model AUC = 0.787. When Tokat was excluded (*n* = 237), PNI OR = 0.909 (*p* = 0.004) with AUC = 0.774. When Artvin was excluded (*n* = 299), PNI OR = 0.890 (*p* < 0.001) with AUC = 0.792. ESR retained significance across all three iterations (all *p* < 0.03). The consistency of effect estimates across these geographically and demographically distinct subsets—despite differences in center-level treatment failure rates (Balıkesir 5.0%, Tokat 7.6%, Artvin 8.9%)—supports the generalizability of the model within the Turkish endemic setting.

## 4. Discussion

The present three-center investigation introduces, to the best of our knowledge, the first internally validated prediction model—implemented as a LASSO-derived nomogram—for focal complications in brucellosis. A structured literature query of PubMed/MEDLINE, Scopus, and Web of Science (conducted January 2026; search string: brucellosis AND [nomogram OR prediction model OR clinical prediction rule] AND [focal complication OR spondylitis OR relapse]) yielded no previously validated prognostic models for this endpoint. The model integrates PNI, ESR, CRP, chronic disease stage, and hypertension into a bedside tool that demonstrated robust discrimination (optimism-corrected C-statistic 0.762), adequate calibration (slope 0.894, Hosmer–Lemeshow *p* = 0.857), and meaningful clinical net benefit spanning a broad range of decision thresholds. These findings represent a significant methodological and clinical advance over existing literature, which has been limited to associative biomarker studies without prediction model development or validation.

The identification of PNI as the single most important predictor—both by LASSO standardized coefficient magnitude and by univariate effect size—is noteworthy. PNI integrates serum albumin and lymphocyte count, and may reflect the intersection of nutritional and immunological status, both of which have been independently associated with adverse outcomes in infectious diseases [16,17]. The cytokine milieu in brucellosis—characterized by elevated TNF-α, IL-6, and IFN-γ levels [31]—may further modulate the nutritional-immunological axis captured by PNI. Albumin reflects hepatic synthetic capacity and nutritional adequacy, whereas lymphocytes orchestrate the adaptive immune defense against intracellular organisms including *Brucella* [18,19]. However, the present retrospective data do not establish whether PNI mechanistically contributes to focal disease development or merely serves as a prognostic marker of the host’s inflammatory and nutritional state. This distinction between predictive utility and causal importance is fundamental and warrants investigation through prospective mechanistic studies. PNI has demonstrated prognostic value in tuberculosis [16] and COVID-19 [17], but no prior study has evaluated its predictive role in brucellosis.

Equally notable is the elimination of NLR, PLR, CAR, SIRI, and other purely hematological ratios by LASSO for the focal complication outcome. Although SII [32] and SIRI [33] have demonstrated prognostic value in oncological settings, and CAR has predicted outcomes in severe sepsis [34,35] and acute medical admissions [36], these indices were rendered redundant in our brucellosis model once PNI, ESR, and CRP were included. This hierarchical finding suggests that nutritional–inflammatory composite indices (PNI) and direct acute-phase markers (CRP, ESR) capture the prognostically relevant information more efficiently than derived hematological ratios. Similar findings have been reported in COVID-19, where PNI outperformed individual hematological ratios for mortality prediction [17]. This has practical implications: clinicians need not calculate complex ratios when PNI, CRP, and ESR—all routinely available—provide superior prognostic information.

The clinical utility of the nomogram, quantified by DCA, represents a critical strength. While many biomarker studies report AUC values without translating them into clinical decision-making frameworks, our DCA demonstrates that the nomogram provides net benefit across threshold probabilities of 5–55%. In practical terms, this means that for a clinician who considers enhanced surveillance warranted at risk levels above 15% (a reasonable clinical threshold), the nomogram correctly identifies additional patients who benefit from investigation while avoiding unnecessary interventions in low-risk patients. The superiority of the full nomogram over PNI alone further justifies the multivariable approach. In practical terms, the nomogram could be applied at the time of brucellosis diagnosis using routinely available parameters. For example, a 55-year-old male patient presenting with chronic brucellosis, hypertension, PNI of 38, ESR of 65 mm/h, and CRP of 45 mg/L would receive a total nomogram score of approximately 195 points, corresponding to a predicted probability of approximately 55%, warranting prompt comprehensive imaging (spinal MRI, echocardiography), closer monitoring, and consideration of prolonged therapy. Conversely, a low-risk patient (predicted probability < 12.4%) could be managed with standard protocols, potentially reducing unnecessary investigations.

The substantial incremental value over the conventional clinical model (ΔAUC = 0.175, NRI = 0.857, IDI = 0.167) underscores the importance of integrating laboratory biomarkers into clinical risk assessment. The conventional model, relying solely on demographics and comorbidities, achieved only modest discrimination (AUC = 0.607), highlighting the inadequacy of clinical judgment alone for focal complication prediction. The addition of PNI, ESR, and CRP—all obtainable from routine admission blood tests at no additional cost—transforms prediction accuracy meaningfully.

Our finding that chronic disease stage was the dominant predictor of relapse (OR = 8.492) is consistent with previous reports [3,9]. Chronic disease was defined as symptom duration exceeding 8 weeks at presentation, in accordance with the widely accepted classification by Franco et al. [2]. The 27.0% relapse rate among chronic-stage patients underscores the need for prolonged treatment and close follow-up in this subgroup [13]. The strong association of male sex with relapse (OR = 3.150, *p* = 0.08) approached significance and aligns with established literature [4,10].

The emergence of hypertension as an independent predictor of focal complications (OR = 2.123) is an unexpected finding. We hypothesize that hypertension may serve as a surrogate marker for microvascular dysfunction, endothelial impairment, or comorbidity burden, conditions that could facilitate bacterial seeding in end organs; however, this interpretation is speculative and should be considered hypothesis-generating. Alternatively, hypertension may act as a proxy for age-related comorbidity or subclinical cardiovascular disease not captured by our predictor set. Confirmatory studies with detailed vascular assessments are needed to clarify this association. Hypertension has been linked to adverse outcomes in other infectious diseases, and it is possible that the association in brucellosis reflects shared pathophysiological pathways rather than a brucellosis-specific mechanism.

Although no previously validated prediction model exists for focal complications in brucellosis, our findings should be contextualized alongside existing risk factor analyses. Demirdal and Sen [11] identified older age, back pain, and elevated ESR as predictors of focal involvement, while Zhang et al. [12] reported male sex, arthralgia, and platelet count as independent risk factors in a Chinese cohort. Copur and Sayili [30] identified CRP, ESR, and disease duration as predictors of focal involvement and bacteremia, and Dadar et al. [3] reported blood culture positivity, symptom duration, and inadequate initial therapy as relapse/focal complication risk factors—findings largely concordant with our LASSO-selected predictors. However, these studies employed conventional regression without penalized variable selection, formal model development, or clinical utility assessment (DCA, NRI/IDI). Our nomogram advances this evidence base by providing individualized probability estimates with quantified discrimination and calibration.

### 4.1. Methodological Considerations

Several analytical decisions warrant elaboration. We opted for LASSO over traditional stepwise methods because the L1 penalty jointly selects predictors and attenuates their coefficients, thereby limiting overfitting—an advantage particularly relevant in datasets of moderate size [37]. However, our approach was not purely data-driven: after LASSO screening, we applied clinician-guided refinement based on prespecified criteria (correlation thresholds, clinical reliability, and coefficient magnitude). This hybrid LASSO-informed, clinician-refined strategy, while clinically pragmatic, introduces investigator judgment that may affect reproducibility. Replication with independent datasets is needed to confirm the selected predictor set. Second, the two-stage approach of LASSO selection followed by unpenalized logistic regression refit is common but may produce anti-conservative confidence intervals and *p*-values due to post-selection inference bias. To partially address this, we report shrinkage-adjusted odds ratios derived by applying the bootstrap-estimated calibration slope (0.894) as a uniform shrinkage factor to the linear predictor coefficients. The calibration slope below 1.0 confirms mild overfitting, which is expected given the sample size and underscores the importance of external validation before clinical implementation. We chose the two-stage refit approach over retaining the penalized LASSO model directly because nomogram construction requires explicit regression coefficients with interpretable units, which penalized estimates do not straightforwardly provide; furthermore, the shrinkage correction applied post hoc achieves a similar effect to direct penalization for calibration purposes. Third, bootstrap internal validation (1000 iterations) was preferred over split-sample approaches, as it provides more efficient optimism estimation in moderate-sized datasets [26,27].

### 4.2. Strengths and Limitations

Key strengths of this work encompass its multicenter architecture across three geographically separated hospitals, the use of LASSO penalization among 32 candidate predictors to achieve data-driven model parsimony, full compliance with the TRIPOD reporting checklist, a multifaceted validation strategy (discrimination, calibration, decision-curve analysis, reclassification metrics, and center-level sensitivity analyses), and the translation of results into a nomogram that relies exclusively on routine laboratory measurements.

Limitations include: (1) the retrospective design, precluding causal inference and introducing potential detection bias—patients with more pronounced inflammatory markers may have undergone more extensive imaging, potentially inflating associations between inflammatory biomarkers and detected focal disease; our nomogram therefore predicts detected focal complications conditional on prevailing diagnostic practices rather than true latent focal disease. However, all three centers followed similar clinical protocols based on Turkish national guidelines, in which imaging is recommended for all brucellosis patients with relevant symptoms regardless of laboratory findings, and the most common focal complication—spondylitis (47.8%)—typically presents with prominent back pain that triggers imaging independent of inflammatory marker levels; (2) the absence of external validation—while leave-one-center-out analyses demonstrated internal geographic robustness, all three centers share the same national healthcare context and similar diagnostic practices, which does not substitute for true geographic or temporal external validation; prospective multicenter validation in geographically diverse endemic settings is the essential next step, and the model should be considered preliminary until externally confirmed; (3) temporal validation was not performed despite the 10-year study period (2015–2025), because the centers contributed data over partially overlapping but non-identical periods, and a temporal split would have reduced the development set below the minimum required sample size for the final model; (4) the exclusion of blood culture status (26.5% missing) may limit the model’s comprehensiveness, as bacteremia has been associated with disease severity in prior literature—future prospective studies should ensure systematic blood culture collection; (5) the composite focal complication endpoint pools pathophysiologically heterogeneous manifestations, and predictors of osteoarticular disease may not perform identically for neurologic or cardiac complications—however, individual subtypes had insufficient events for separate modeling; (6) the screening-stage EPV of 2.9 is low, and although LASSO penalization mitigates instability, the formal a priori sample size calculation was not performed; (7) the hybrid LASSO-informed, clinician-refined variable selection strategy introduces investigator discretion that may limit reproducibility; and (8) the secondary outcome analyses (treatment failure, relapse) are exploratory given their low event counts and should not be used for clinical decision-making without independent confirmation.

## 5. Conclusions

We constructed and internally validated what appears to be the first LASSO-derived nomogram targeting focal complications in brucellosis. With PNI serving as the principal predictor—a finding not previously reported in this disease—alongside ESR, CRP, chronic stage, and hypertension, the instrument achieved satisfactory discrimination (optimism-corrected C-statistic 0.762) and well-calibrated probability estimates. The nomogram may facilitate early risk stratification using routinely available clinical and laboratory parameters. However, this internally validated model should be considered preliminary pending external validation in geographically diverse endemic populations. The observed associations should not be interpreted as causal, and this study should be regarded as hypothesis-generating regarding the biological mechanisms underlying PNI’s predictive role in brucellosis. Prospective multicenter external validation is the essential next step before clinical implementation can be recommended.

## Figures and Tables

**Table 1:** Baseline characteristics stratified by focal complication status.

Variable	Non-Focal (*n* = 263)	Focal (*n* = 92)	*p*
Age (years), mean ± SD	47.7 ± 15.4	50.0 ± 15.9	0.226
Male sex	193 (73.4)	68 (73.9)	1.000
Rural residence	213 (81.0)	82 (89.1)	0.103
Hypertension	29 (11.0)	19 (20.7)	0.032 *
Chronic disease stage	22 (8.4)	15 (16.3)	0.052
CRP (mg/L)	18.8 (10.8–32.1)	30.8 (16.0–55.1)	<0.001 *
ESR (mm/h)	20 (13–30)	30 (22–40)	<0.001 *
Albumin (g/L)	41 (38–43)	39 (34–41)	<0.001 *
Neutrophil (/μL)	4080 (3070–5100)	4505 (3618–5703)	0.002 *
Lymphocyte (/μL)	2380 ± 806	2115 ± 589	0.004 *
Platelet (×10^3^/μL)	245 (198–284)	266 (209–310)	0.026 *
**NLR**	1.69 (1.27–2.45)	2.30 (1.63–3.22)	<0.001 *
**PLR**	105 (78–138)	120 (91–169)	0.002 *
**SII (×10^3^)**	392,488 (271,000–615,000)	566,500 (374,000–872,000)	<0.001 *
**PNI, mean ± SD**	52.6 ± 5.8	48.7 ± 5.3	<0.001 *
**CAR**	0.45 (0.27–0.80)	0.84 (0.41–1.41)	<0.001 *

Values are median (IQR), mean ± SD, or *n* (%). * *p* < 0.05. NLR, neutrophil-to-lymphocyte ratio; PLR, platelet-to-lymphocyte ratio; SII, systemic immune-inflammation index (×10^3^); CAR, CRP-to-albumin ratio; PNI, prognostic nutritional index.

**Table 2:** LASSO-informed multivariate logistic regression model for focal complications (nomogram model).

Variable	β	OR	95% CI	*p*-Value	Shrinkage-Adjusted OR
Age (years)	−0.004	0.996	0.979–1.014	0.662	0.996
Male sex	0.121	1.128	0.601–2.118	0.708	1.114
**PNI (per unit)**	−0.104	0.901	0.857–0.948	**<0.001**	0.911
**ESR (per mm/h)**	0.029	1.030	1.013–1.047	**<0.001**	1.027
**CRP (per mg/L)**	0.013	1.014	1.005–1.022	**0.002**	1.012
**Chronic disease stage**	0.909	2.481	1.107–5.559	**0.027**	2.246
**Hypertension**	0.753	2.123	1.026–4.393	**0.043**	1.956

**Table 3:** Risk stratification by model-predicted probability tertiles.

Risk Group	Predicted Probability	*n*	Focal Events	Rate (%)	Fold vs. Low
Low	<12.4%	118	8	6.8	1.0 (ref)
Medium	12.4–27.8%	119	27	22.7	3.3
High	>27.8%	118	57	48.3	7.1
*p* for trend				**<0.001**	

## Data Availability

The datasets generated and analyzed during the current study are available from the corresponding author upon reasonable request.

## Supplementary Materials

The following supporting information can be downloaded at: https://www.mdpi.com/article/10.3390/jcm15062180/s1, Figure S1: Participant flow diagram; Figure S2: Correlation matrix of candidate predictors; Figure S3: Forest plot of the LASSO-selected multivariate logistic regression model; Table S1: LASSO variable selection results; Table S2: Comparison of complete-case and MICE-imputed model coefficients; Table S3: Incremental value of the nomogram over conventional clinical model; Table S4: Leave-one-center-out sensitivity analyses; Table S5: TRIPOD Checklist; Table S6: STROBE Checklist.

## Abbreviations

AIC	Akaike Information Criterion
ALT	alanine aminotransferase
AST	aspartate aminotransferase
AUC	area under the receiver operating characteristic curve
CAR	CRP-to-albumin ratio
CI	confidence interval
CRP	C-reactive protein
DCA	decision curve analysis
EPV	events per variable
ESR	erythrocyte sedimentation rate
IDI	integrated discrimination improvement
IFN-γ	interferon gamma
IL-6	interleukin-6
IQR	interquartile range
LASSO	Least Absolute Shrinkage and Selection Operator
MCV	mean corpuscular volume
MICE	multiple imputation by chained equations
MLR	monocyte-to-lymphocyte ratio
MRI	magnetic resonance imaging
NLR	neutrophil-to-lymphocyte ratio
NRI	net reclassification improvement
OR	odds ratio
PLR	platelet-to-lymphocyte ratio
PNI	Prognostic Nutritional Index
ROC	receiver operating characteristic
SAT	standard tube agglutination
SD	standard deviation
SII	systemic immune–inflammation index
SIRI	systemic inflammation response index
STROBE	Strengthening the Reporting of Observational Studies in Epidemiology
TNF-α	tumor necrosis factor alpha
TRIPOD	Transparent Reporting of a multivariable prediction model for Individual Prognosis Or Diagnosis
VIF	variance inflation factor
WBC	white blood cell
