# Supplementary material for: A LASSO-Based Nomogram for Predicting Focal Complications in Brucellosis: A Multicenter Retrospective Cohort Study
Source: J Clin Med. 2026 Mar 12;15(6):2180. doi: 10.3390/jcm15062180 (PMC13027353; doi:10.3390/jcm15062180)
Supplement: Supplementary file 1 [file jcm-15-02180-s001.zip › Supplementary_Materials.pdf]

SUPPLEMENTARY MATERIAL

A LASSO-Based Nomogram for Predicting Focal Complications in Brucellosis: A Multicenter Retrospective Cohort Study

Table S1. LASSO variable selection results: non-zero coefficients from 32 candidate predictors at λ.min, ranked by absolute standardized β.

Twelve variables with zero coefficients (eliminated by LASSO) are not shown: age, sex, cardiovascular disease, fever, animal contact, dairy consumption, PLR, NLR, CAR, SIRI, albumin, lymphocyte count.

Variable	Standardized β at λ.min	Retained in Final Model (Rationale)
PNI	−0.677	Yes
ESR (mm/h)	0.489	Yes
Neutrophil count	0.371	No (subsumed by PNI/SII)
Platelet count	0.321	No (subsumed by SII)
CRP (mg/L)	0.301	Yes
SII	0.287	No (r = 0.893 with NLR)
Chronic disease stage	0.243	Yes
MLR	0.198	No (redundant with SIRI)
AST (U/L)	0.157	No (r = 0.735 with ALT)
Weight loss	0.139	No (subjective symptom)
Night sweats	0.128	No (subjective symptom)
Hypertension	0.122	Yes
ALT (U/L)	0.108	No (r = 0.735 with AST)
Hemoglobin (g/dL)	−0.098	No (marginal β)
Myalgia	0.087	No (subjective symptom)
MCV (fL)	−0.076	No (marginal β)
Monocyte count	0.065	No (subsumed by MLR/SIRI)
Rural residence	0.054	No (marginal β)
Fatigue	0.042	No (subjective symptom)
Diabetes	0.031	No (marginal β)
WBC	0.025	No (r = 0.931 with neutrophil)
Arthralgia	0.018	No (marginal β)

Table S2. Comparison of model coefficients between complete-case analysis and multiple imputation by chained equations (MICE).

MICE specification: 20 imputations, 50 iterations, predictive mean matching for continuous variables, logistic regression for binary variables. All 32 candidate predictors plus the outcome were included in the imputation model. OR, odds ratio; CI, confidence interval; AUC, area under the receiver operating characteristic curve; PNI, prognostic nutritional index; ESR, erythrocyte sedimentation rate; CRP, C-reactive protein.

Variable	Complete-Case OR (95% CI)	MICE-Imputed OR (95% CI)	Difference
PNI (per unit)	0.901 (0.857–0.948)	0.901 (0.856–0.949)	0.000
ESR (per mm/h)	1.030 (1.013–1.047)	1.030 (1.013–1.047)	0.000
CRP (per mg/L)	1.014 (1.005–1.022)	1.013 (1.004–1.022)	−0.001
Chronic stage	2.481 (1.107–5.559)	2.497 (1.114–5.596)	+0.016
Hypertension	2.123 (1.026–4.393)	2.142 (1.035–4.432)	+0.019
Age (per year)	0.996 (0.979–1.014)	0.996 (0.979–1.014)	0.000
Male sex	1.128 (0.601–2.118)	1.118 (0.596–2.098)	−0.010
Model AUC	0.782	0.781	−0.001

Note: Differences in odds ratios between complete-case and MICE analyses were negligible (≤0.019 for all variables), supporting the robustness of the complete-case approach given the minimal missingness (<2% for laboratory variables). Blood culture status (26.5% missing) was excluded from both analyses due to non-random missingness patterns.

Table S3. Incremental value of the nomogram over the conventional clinical model (age, sex, chronic stage, hypertension, diabetes, fever).

Category-free NRI may overestimate reclassification improvement; IDI provides a more conservative estimate. AIC = Akaike Information Criterion; NRI = net reclassification improvement; IDI = integrated discrimination improvement.

Metric	Conventional Model	Nomogram	Difference	p-Value
AUC (95% CI)	0.607 (0.542–0.672)	0.782 (0.726–0.838)	0.175	<0.001
AIC	410.6	355.0	−55.6	—
NRI (total)	—	—	0.857	<0.001
NRI (events)	—	—	0.391	—
NRI (non-events)	—	—	0.466	—
IDI	—	—	0.167	<0.001

Table S4. Leave-one-center-out sensitivity analyses for the focal complication nomogram.

Each row excludes one center and refits the model on the remaining two centers. PNI and ESR retained significance across all iterations.

Analysis	PNI OR (95% CI)	p	ESR OR (95% CI)	p	AUC
Excl. Balıkesir (n = 174)	0.895 (0.839–0.956)	0.002	1.032 (1.011–1.053)	0.003	0.787
Excl. Tokat (n = 237)	0.909 (0.857–0.964)	0.004	1.028 (1.008–1.048)	0.006	0.774
Excl. Artvin (n = 299)	0.890 (0.845–0.938)	<0.001	1.033 (1.015–1.051)	<0.001	0.792

Table S5. TRIPOD Checklist for the brucellosis focal complication nomogram.

D = Development; V = Validation; N/A = Not applicable. TRIPOD = Transparent Reporting of a multivariable prediction model for Individual Prognosis Or Diagnosis.

Section/Topic	Item	D/V	Reported (Location in Manuscript)
Title	1	D	Title identifies as prediction model study
Abstract	2	D	Structured abstract: Background, Methods, Results, Conclusions (306 words)
Background/Objectives	3a	D	Medical context and rationale (Introduction, para 1–3)
	3b	D	Three specific objectives (Introduction, final para)
Source of data	4a	D	Multicenter retrospective cohort, 3 centers, 2015–2025 (Section 2.1)
	4b	D	Key dates specified (Section 2.1)
Participants	5a	D	Eligibility criteria: age ≥18, confirmed brucellosis, exclusions (Section 2.2)
	5b	D	Consecutive eligible patients (Section 2.2)
Outcome	6a	D	Focal complication defined with organ manifestations (Section 2.4)
	6b	D	Blinding: N/A (retrospective)
Predictors	7a	D	32 candidate predictors prespecified (Section 2.3)
	7b	D	All predictor assessments defined including composite index formulas (Section 2.3)
Sample size	8	D	Post-hoc EPV: screening 2.9, final 13.1 (Section 2.2)
Missing data	9	D	Complete-case primary; MICE sensitivity (Section 2.5)
Statistical analysis	10a	D	LASSO with 10-fold CV described (Section 2.5)
	10b	D	Full model: 7 parameters including a priori covariates
	10d	D	Bootstrap 1000× for internal validation (Section 2.5)
Risk groups	11	D	Tertile-based risk groups (Table 3, Section 3.8)
Development vs validation	12	D	Type 1b: development with internal validation (bootstrap)
Flow of participants	13a	D	Participant flow diagram (Figure S1)
	13b	D	355 included; follow-up described (Section 3.1)
Characteristics	14a	D	Baseline characteristics by outcome (Table 1)
	14b	D	Events: 92 focal, 22 TF, 26 relapse, 46 overall TF
Model development	15a	D	LASSO selection and multivariate logistic regression (Section 3.3–3.4)
	15b	D	Full model via nomogram (Figure 2) and Table 2
Model performance	16	D	C-statistic 0.782/0.762, calibration slope 0.894, Brier 0.154 (Section 3.5)
Model updating	17	N/A	Not applicable (development study)
Discussion	18	D	Results in context of existing evidence (Section 4)
Limitations	19a	D	Seven limitations discussed (Section 4.2)
	19b	D	Overfitting, no external validation, missing data addressed
Implications	20	D	DCA for clinical utility; external validation as next step
Supplementary	21	D	Full LASSO list (Table S2), sensitivity analyses (Table S4)
Funding	22	D	No external funding (Funding statement)

Table S6. STROBE Checklist for cohort studies.

STROBE = Strengthening the Reporting of Observational Studies in Epidemiology. Items adapted from von Elm et al. (2008). ✓ = reported.

Item	No.	Reported (Location)
Title and abstract	1	(a) Study design in title ✓; (b) Informative, balanced abstract (306 words) ✓
Background/rationale	2	Scientific background and rationale (Introduction, para 1–3) ✓
Objectives	3	Three specific objectives stated (Introduction, final para) ✓
Study design	4	Retrospective multicenter cohort study (Section 2.1) ✓
Setting	5	Three centers in Türkiye; January 2015–December 2025 (Section 2.1) ✓
Participants	6	(a) Eligibility criteria, sources, selection methods (Section 2.2) ✓
Variables	7	32 candidate predictors defined with composite index formulas (Section 2.3) ✓
Data sources/measurement	8	Data sources and assessment methods described (Sections 2.3–2.4) ✓
Bias	9	LASSO for overfitting reduction; bootstrap validation; leave-one-center-out; MICE sensitivity (Sections 2.5, 3.10) ✓
Study size	10	355 patients from 3 centers; post-hoc EPV assessment (Section 2.2) ✓
Quantitative variables	11	Continuous variable handling; composite index calculations (Section 2.3) ✓
Statistical methods	12	(a–e) LASSO, logistic regression, bootstrap, DCA, NRI/IDI; missing data; sensitivity analyses (Section 2.5) ✓
Participants	13	(a) Flow diagram (Figure S1); (b) N/A (consecutive enrollment) ✓
Descriptive data	14	(a) Demographics/characteristics (Table 1); (b) Missing data: <2% labs, 26.5% blood culture (Section 2.5) ✓
Outcome data	15	Events: 92 focal, 22 TF, 26 relapse, 46 overall TF (Sections 3.2, 3.9) ✓
Main results	16	(a) Unadjusted/adjusted estimates (Tables 1–2); (b) CI and p-values; (c) Tertile categories (Table 3) ✓
Other analyses	17	DCA, NRI/IDI, leave-one-center-out, MICE (Sections 3.6–3.10) ✓
Key results	18	Key results summarized referencing objectives (Discussion, para 1) ✓
Limitations	19	Seven specific limitations with bias direction (Section 4.2) ✓
Interpretation	20	Cautious interpretation consistent with results and limitations (Section 4) ✓
Generalisability	21	External validity discussed; Turkish endemic setting (Sections 3.10, 4.2) ✓
Funding	22	No external funding (Funding statement) ✓

Supplementary Figure Captions

Figure S1. Participant flow diagram. Of 412 patients screened across three centers (Balıkesir University Hospital, Tokat Turhal State Hospital, and Artvin State Hospital) between January 2015 and December 2025, 57 were excluded (incomplete records n = 23, concurrent malignancy n = 8, immunosuppressive therapy n = 7, chronic liver disease n = 6, pregnancy n = 5, hematological malignancy n = 4, age <18 years n = 4), yielding 355 patients included in the analysis. Center-specific enrollment: Balıkesir n = 181, Tokat n = 118, Artvin n = 56. LASSO variable selection from 32 candidate predictors identified 7 for the final nomogram model with bootstrap internal validation (×1000). Focal complications occurred in 92 patients (25.9%); secondary outcomes included treatment failure (n = 22, 6.2%), relapse (n = 26, 7.3%), and overall treatment failure (n = 46, 13.0%).

Figure S2. Pairwise Pearson correlation matrix of 32 candidate predictors (n = 355). The lower triangle displays Pearson correlation coefficients; values ≥|0.50| are annotated with numeric labels. Color scale ranges from −1.0 (blue) to +1.0 (dark red). Variables are grouped by category: demographics (age, sex, rural residence), disease stage (chronic stage), comorbidities (diabetes, hypertension, cardiovascular disease), symptoms (fever, night sweats, arthralgia, myalgia, fatigue, weight loss), laboratory parameters (CRP, ESR, albumin, WBC, neutrophil, lymphocyte, monocyte, hemoglobin, platelet, MCV, ALT, AST), and composite inflammatory/nutritional indices (NLR, PLR, MLR, SII, SIRI, PNI, CAR). Notable high correlations include: neutrophil–WBC (r = 0.93), SII–NLR (r = 0.89), CAR–CRP (r = 0.98), AST–ALT (r = 0.74), and PNI–albumin (r = 0.76). These intercorrelations among composite indices and their constituent parameters supported the LASSO-based elimination of redundant predictors.

Figure S3. Forest plot of multivariate logistic regression results for LASSO-selected predictors of focal complications. Each point represents the adjusted odds ratio (OR) on a logarithmic scale, with horizontal lines indicating 95% confidence intervals (CI). Red points with asterisks (*) denote statistically significant predictors ($p < 0.05$): CRP (OR = 1.014, 95% CI: 1.005–1.022), ESR (OR = 1.030, 95% CI: 1.013–1.047), PNI (OR = 0.901, 95% CI: 0.857–0.948), hypertension (OR = 2.123, 95% CI: 1.026–4.393), and chronic stage (OR = 2.481, 95% CI: 1.107–5.559). Gray points indicate non-significant a priori covariates: male sex (OR = 1.128, 95% CI: 0.601–2.118) and age (OR = 0.996, 95% CI: 0.979–1.014). The vertical dashed line at OR = 1.0 represents no effect.
