# Supplementary figures and images for: A LASSO-Based Nomogram for Predicting Focal Complications in Brucellosis: A Multicenter Retrospective Cohort Study
Source: J Clin Med. 2026 Mar 12;15(6):2180. doi: 10.3390/jcm15062180 (PMC13027353; doi:10.3390/jcm15062180)

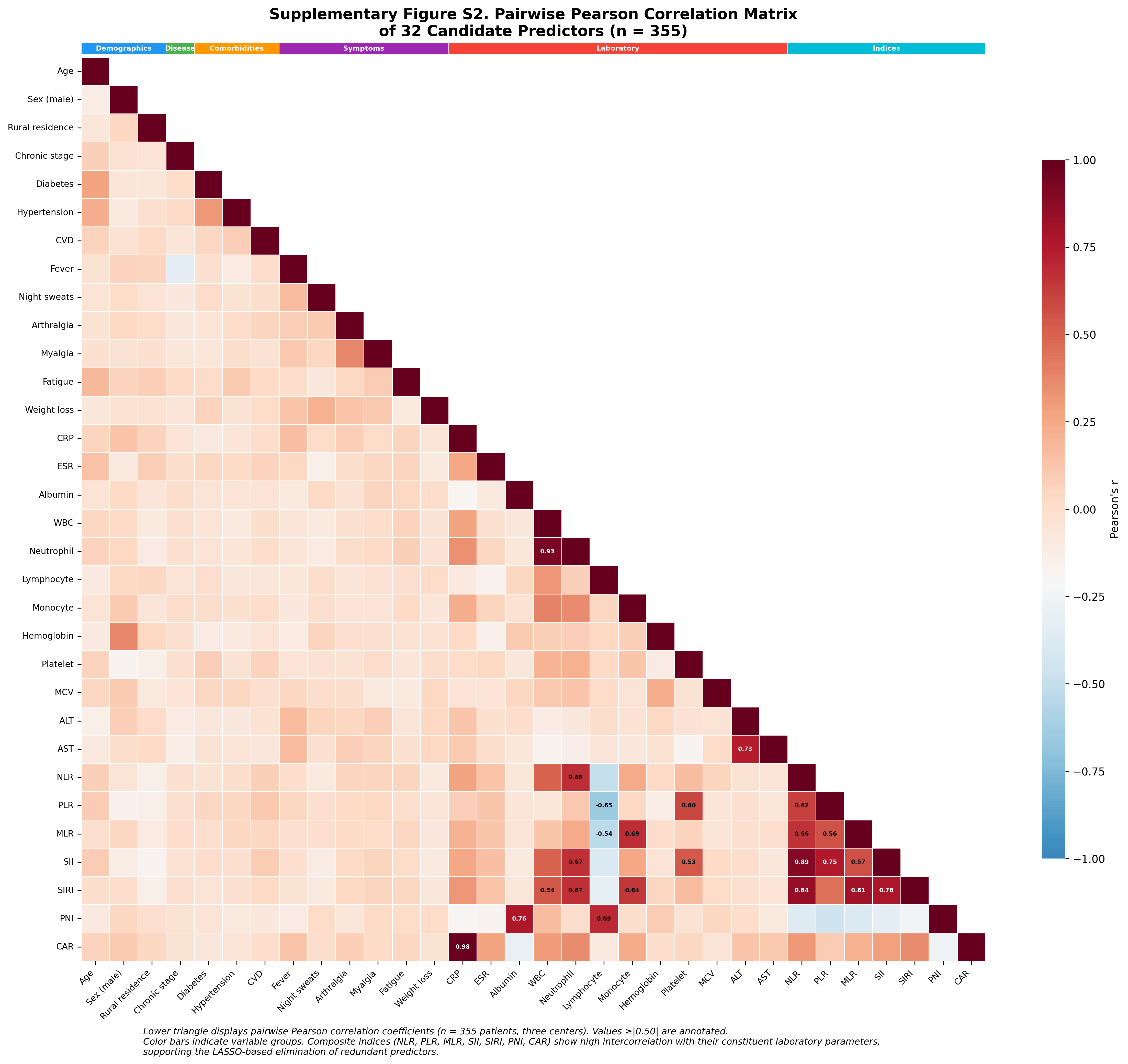

Supplement: Supplementary file 1 [file jcm-15-02180-s001.zip › Figure_S2_Correlation_Matrix.png]

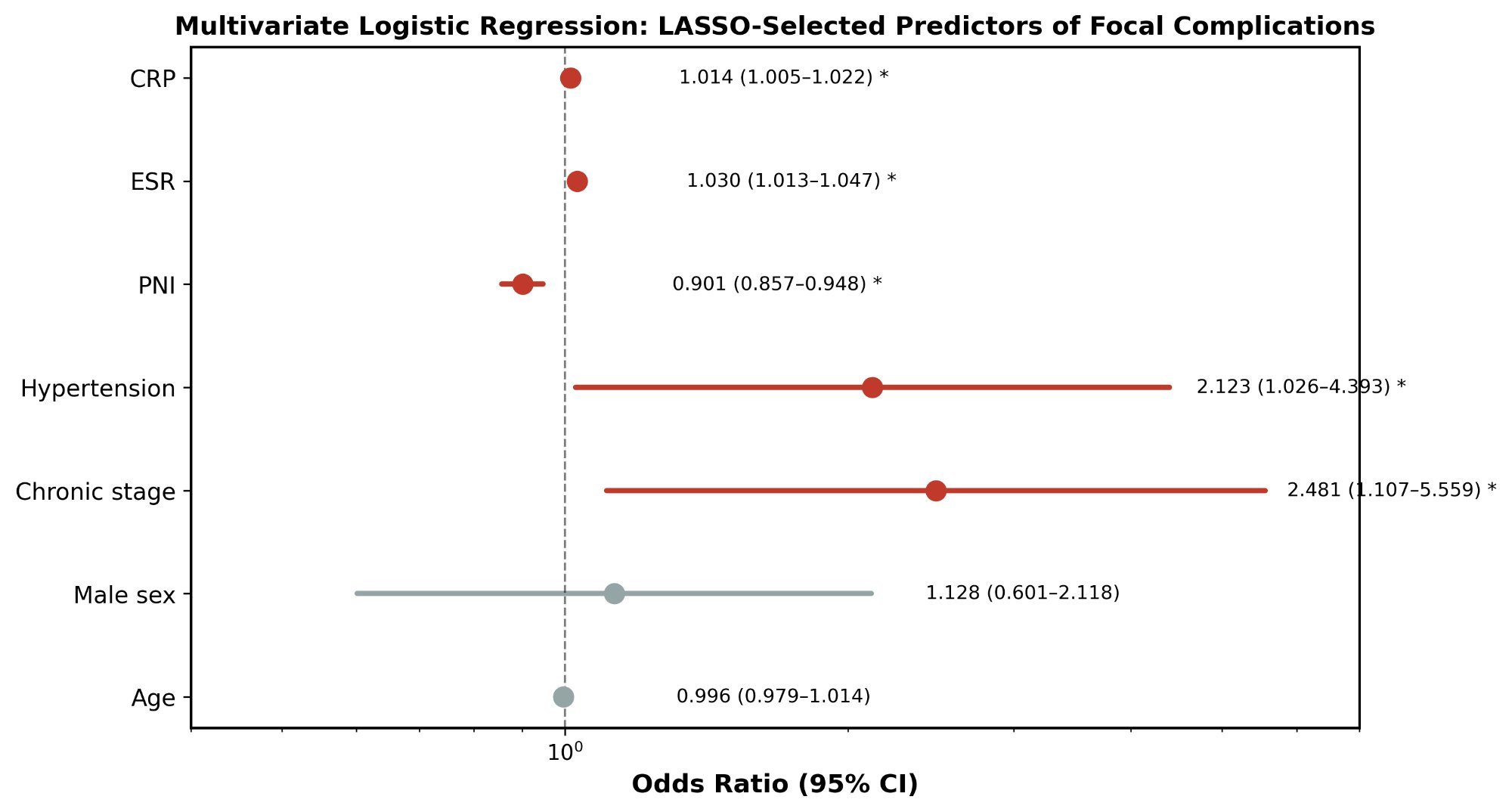

Supplement: Supplementary file 1 [file jcm-15-02180-s001.zip › Figure_S3_Forest_Plot.png]

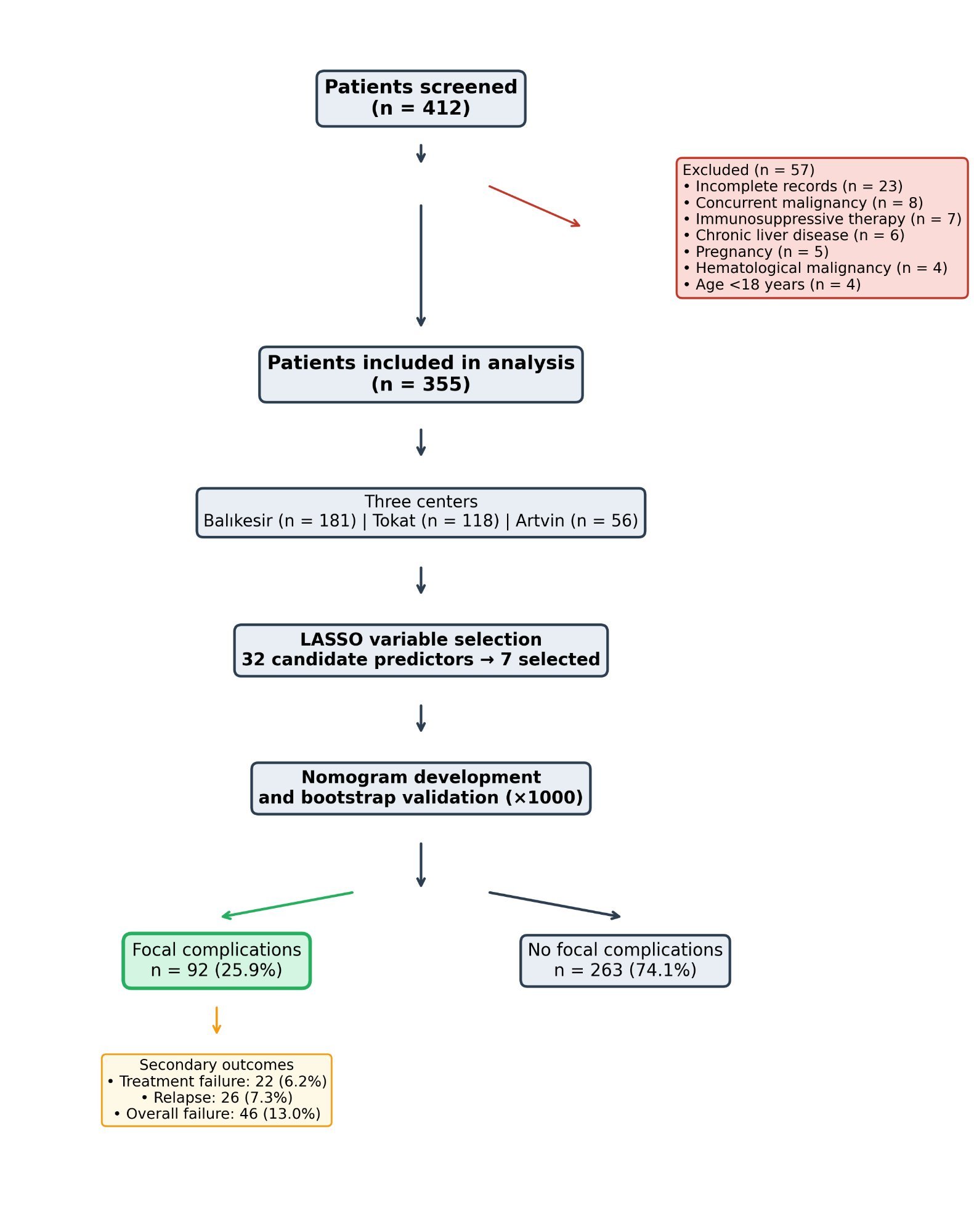

Supplement: Supplementary file 1 [file jcm-15-02180-s001.zip › Figure_S1_Flow_Diagram.png]
